# Genetic Analysis of Multiple Myeloma Identifies Cytogenetic Alterations Implicated in Disease Complexity and Progression

**DOI:** 10.3390/cancers13030517

**Published:** 2021-01-29

**Authors:** Can Li, Erik B. Wendlandt, Benjamin Darbro, Hongwei Xu, Gregory S. Thomas, Guido Tricot, Fangping Chen, John D. Shaughnessy, Fenghuang Zhan

**Affiliations:** 1 Myeloma Center, Department of Internal Medicine, Winthrop P. Rockefeller Cancer Institute, University of Arkansas for Medical Sciences, Little Rock, AR 72205, USA; cli2@uams.edu (C.L.); HXu@uams.edu (H.X.); GJTricot@uams.edu (G.T.); JDShaughnessy@uams.edu (J.D.S.J.); 2 Department of Hematology, Xiangya Hospital, Central South University, Changsha 410008, China; xychenfp@csu.edu.cn; 3 Department of Internal Medicine, University of Iowa, Iowa City, IA 52242, USA; ewendlandt@idtdna.com (E.B.W.); gregory-thomas@uiowa.edu (G.S.T.); 4 Cytogenetics and Molecular Laboratory, Carver College of Medicine, University of Iowa, Iowa City, IA 52242, USA; benjamin-darbro@uiowa.edu

**Keywords:** multiple myeloma, copy number variations, gene expression profiles, cytogenetics, protein network signatures

## Abstract

**Simple Summary:**

Multiple myeloma (MM) is the second most common hematological neoplasia with a high incidence in elderly populations. The disease is characterized by a severe chaos of genomic abnormality. Comprehensive examinations of myeloma cytogenetics are needed for better understanding of MM and potential application to the development of novel therapeutic regiments. Here we utilized gene expression profiling and CytoScan HD genomic arrays to investigate molecular alterations in myeloma leading to disease progression and poor clinical outcomes. We demonstrates that genetic abnormalities within MM patients exhibit unique protein network signatures that can be exploited for implementation of existing therapies targeting key pathways and the development of novel therapeutics.

**Abstract:**

Multiple myeloma (MM) is a genetically heterogeneous disease characterized by genomic chaos making it difficult to distinguish driver from passenger mutations. In this study, we integrated data from whole genome gene expression profiling (GEP) microarrays and CytoScan HD high-resolution genomic arrays to integrate GEP with copy number variations (CNV) to more precisely define molecular alterations in MM important for disease initiation, progression and poor clinical outcome. We utilized gene expression arrays from 351 MM samples and CytoScan HD arrays from 97 MM samples to identify eight CNV events that represent possible MM drivers. By integrating GEP and CNV data we divided the MM into eight unique subgroups and demonstrated that patients within one of the eight distinct subgroups exhibited common and unique protein network signatures that can be utilized to identify new therapeutic interventions based on pathway dysregulation. Data also point to the central role of 1q gains and the upregulated expression of ANP32E, DTL, IFI16, UBE2Q1, and UBE2T as potential drivers of MM aggressiveness. The data presented here utilized a novel approach to identify potential driver CNV events in MM, the creation of an improved definition of the molecular basis of MM and the identification of potential new points of therapeutic intervention.

## 1. Introduction

Multiple myeloma (MM) is a genetically complex clonal plasma cell malignancy with an increased incidence in aged populations, accounting for 1% of all diagnosed neoplastic diseases and 13% of hematologic cancers [1,2,3]. Myeloma is characterized by accumulation of malignant plasma cells in the bone marrow of the appendicular skeleton and manifests itself in a multistep process from the asymptomatic monoclonal gammopathy of undetermined significance (MGUS) to smoldering multiple myeloma (SMM) to symptomatic MM with increased plasma cell infiltration an attendant anemia, immunosuppression, kidney failure and lytic bone disease [4,5,6]. Tumor cells often exhibit chaotic metaphase karyotypes with numerous gross chromosome translocations, gains and losses, deletions and amplifications. Indeed, cataloging such karyotypic events across hundreds of cases has shown abnormalities are seen in every chromosome [7]. Molecular studies have demonstrated that the genomic chaos present in MM is already present in MGUS. As such, differentiating driver from passenger genetic lesions have been exceedingly difficult. Nevertheless, recurrent changes have been recognized. Approximately 30–40% of cases harbor a t(6;14), t(11;14), t(4;14), t(14;20) or t(14;16) translocation involving the fusion of the immunoglobulin heavy chain locus to CCND1, CCND3, MAFA, MAF, MAFB, FGFR3 and WHSC1 [8,9,10,11,12,13,14,15,16]. Tumor cells often harbor the simultaneous gains of chromosomes 3, 5, 7, 9, 11, 15, 19, and 21 in what has been termed hyperdiploidy is seen in nearly 80% of cases. The 14q translocations, as well as recurrent gains of 1q and amplification of 1q21 as well as deletions of 1p, 6q and 13q are observed in both hyperdiploid and non-hyperdiploid disease. More recently, we and others have created a comprehensive molecular classification of MM showing that the disease can be divided into at least seven subtypes characterized by co-expression of gene clusters, associated with previously defined 14q translocations and hyperdiploidy [17,18].

Copy number variations (CNV) have been recognized as important genomic alterations leading to cancer pathogenesis are defined as segment of DNA longer than 1 kb and containing 90% sequence homology to the parental segment but varies in the number of copies between individuals [19]. Furthermore, CNVs are known to affect higher percentages of DNA than single-nucleotide polymorphism (SNP) variations and CNVs contribute significantly to variation among individuals in gene expression and medically relevant phenotypes [20,21,22]. Gene expression levels have been correlated with CNV as defined by iFISH, cytogenetics and low-resolution SNP arrays [23]. As such GEP can be used to accurately predict the presence of CNVs in purified tumor cells. Specifically, GEP can be used to identify gains of chromosome 1q, 3q, 5q, 7q, 9q, 11q, 15q, 19q, and 21q as well as deletions 1p, 6q and 13q and amplifications of 1q21 [23].

In the current study we aimed to integrate GEP data from U133Plus2.0 microarrays with high-resolution CNV data generated with the CytoScan™ HD Array that includes 2.67 million markers for copy number (CN) analysis, including 750,000 SNP probes and 1.9 million non-polymorphic probes using highly enriched tumor cells from newly diagnosed MM to identify key molecular markers implicated in disease progression and clinical outcome. We found that gains at specific regions on chromosomes 1, 3, 9, 15 and 19 and deletions at regions of chromosomes 8, 13 and 16 are molecular drivers of aggressive disease progression resulting in poor clinical outcomes. We then performed subgroup analysis on myeloma patient samples divided into eight subgroups based on gene expression and cytogenetic markers. We found that, while very similar in chromosomal structure, each subgroup exhibited unique features that stood out from the other subgroups. Furthermore, pathway analysis revealed unique protein interaction networks for each of the subgroups as well as common networks shared between the subgroups. Analysis of the unique protein interaction networks revealed novel drug targets that can be exploited clinically to treat patients fitting into one of the defined profiles. The work presented within offers a new approach for identification and treatment of myeloma based on a small subset of genetic probes derived from high resolution profiling.

## 2. Results

### 2.1. Changes in Gene Expression across the Unique Stages of Myeloma

**Figure 1:**
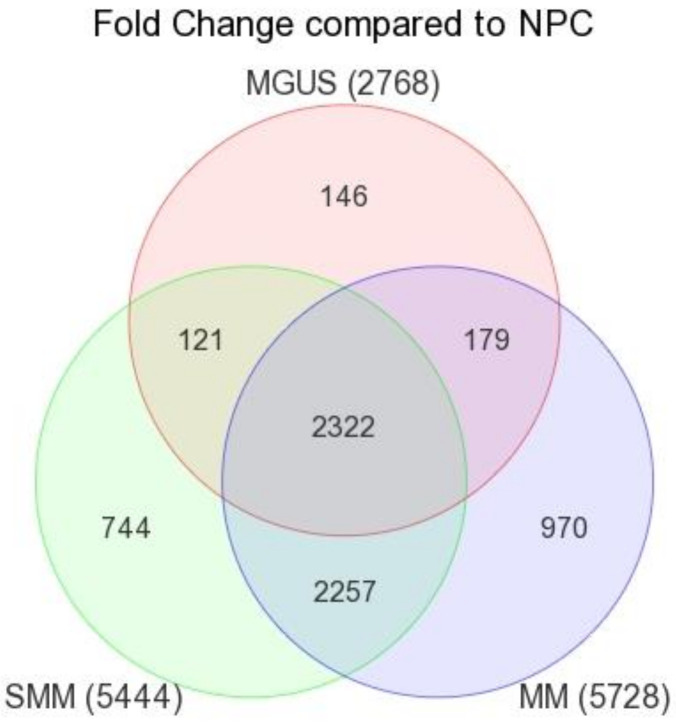
Gene expression analysis of MGUS, SMM and MM samples. Gene expression profiles of CD138-selected plasma cells from the bone marrow from 351 newly diagnosed MM, 22 SMM, 44 MGUS and 22 healthy donors (NPC) were compared. Diagram represents genes with a 1.5 fold or greater difference across each subgroup. Gene expression was compared across the three subgroups to identify overlapping genes within each disease subgroup and genes unique to each subgroup. Significance was determined as *p* < 0.05 with an FDR < 0.05 as determined by a student’s *t*-test.

General understanding of genetic aberrations and changes in gene expression in MM has improved over the last decade with the advent of microarray, high resolution whole genome arrays and sequencing technologies. To explore changes in gene expression across the myeloma spectrum we utilized the TT2 gene expression cohort from the Donna D. and Donald M. Lambert Laboratory of Myeloma Genetics at the University of Arkansas [17]. Significantly altered transcripts for MGUS, SMM and MM, as determined by a fold change of 1.5 fold or greater compared to healthy donors (NPC), were queried to find transcripts common and unique to the three cohorts (Figure 1). Interestingly, there was an increase in total gene dysregulation as the disease progresses from MGUS to MM with MGUS having the lowest number of dysregulated transcripts and the least number of genes unique to the MGUS cohort (Figure 1). Furthermore, MM proved to be the most complex of the three cohorts with the largest number of dysregulated transcripts and the highest number of genes unique to the myeloma cohort (Figure 1). Due to the complex nature of myeloma and the highest proportion of dysregulated transcripts we chose to further examine this group and determine if changes in gene expression are the result of aberrant transcription regulation or due to a change in the copy number at these loci.

### 2.2. Copy Number Analysis of Newly Diagnosed Myeloma Patients

**Figure 2:**
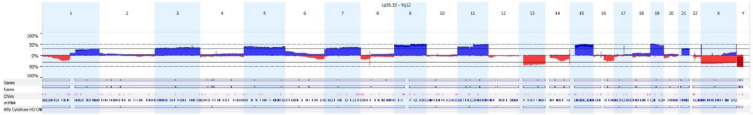
Copy number variation analysis of MM patient samples. Cytoscan HD CNV arrays were performed on CD138-Scheme 97. newly diagnosed MM patients. Copy number variation (CNV) profile was generated for the average change in copy number state across the whole cohort.

Although changes in gene expression play an important role in disease progression and overall pathogenesis, the precise genetic events driving the GEP changes are not well known. CytoScan HD whole genome arrays were performed on purified tumor cells from 97 newly diagnosed myeloma patients to identify changes to the copy number state of each patient (Figure 2). Consistent with previous findings, our analysis identified CNV gains of chromosomes 3, 5, 7, 9, 11, 15, 19, and 21 and loss of chromosomes 13 and 22 [24]. Work by Corre et al. identified two main genetic groups, the first defined by the changes highlighted above whereas the second group is characterized by genetic lesions that affect gains or deletions to subchromosomal material, including gains of 1q and 6p and deletions of 1p, 6q, 8p, 12p, 14q, 16p, 16q, and 20p [25]. Interestingly, results from our analysis not only identified the commonly accepted changes seen in myeloma, but we identified many of the genetic alterations identified Corre et al., including gains to 1q and 6p as well as deletions to 1p, 6q, 8p, 14q, 16q, and 20p. Whole group analysis provides a detailed picture of myeloma genetics but does not take into account the diverse nature of it, therefore an in-depth analysis of the disease is warranted.

### 2.3. Changes in Gene Expression Correlate with Changes in Copy Number State

**Figure 3:**
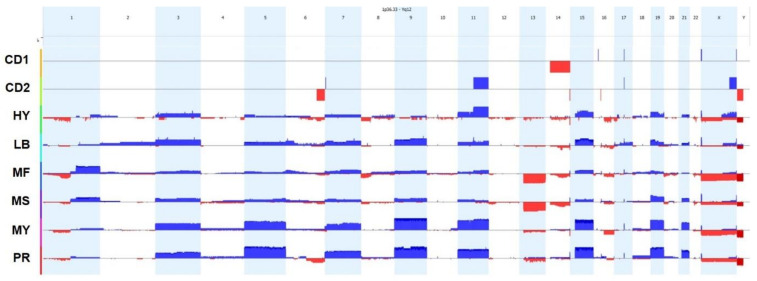
Myeloma Subgroups Exhibit Unique Cytogenetic Signatures Cytogenetic profiles from 97 patient samples were categorized into one of eight defined subgroups based on gene expression profiles. Results represent the average cytogenetic profiles for each of the 8 subgroups.

To further define the genetic makeup of myeloma we chose to divide the samples into seven subgroups based on gene expression profiles of 700 genes (100 per group) linked to recurrent genetic lesions as described in the introduction and previously described [17]. The GEP defined molecular subgroups are as follows: IgH enhancer mediated dysregulation of MAF/MAFB by the t(14;16) or t(14;20) translocation belong to MF group, IgH enhancer superactivation of FGFR3 and/or WHSC1/MMSET by the t(4;14) translocation belong to MS group, the IgH enhancer mediated superactivation of Cyclin D1 or Cyclin D3 by the t(11;14) or t(6;14) translocation with or without concomitant CD20 and VPREB3 expression belong to CD2 group or CD1 group, respectively. Hyperdiploid disease with gains of 3,5,7,9,11,15,19, and 21 without 14q translocations belong to HY group, hyperdiploid disease with gains of 3,5,7,9,15,19, and 21 with also 1q gains, deletion of 13q and absence of 11q gains belong to LB group, and HY, or less frequently LB, with concomitant overexpression of cell cycle and proliferation genes belong to PR group. An eighth group, composed of cases with underlying features of the MF, MS, CD1, CD2, HY and LB subtypes, but also a strong overwhelming myeloid signature, most likely driven by the co-purification of myeloid cells from bone marrow samples with low tumor cell infiltration belong to MY group. GEP data was used to generate a chromosomal map of genetic changes in each sample as an approach to correlate changes in GEP to changes in CNV; results from our subgroup analysis are seen in Figure 3. As anticipated, many of the subgroups exhibited a similar profile to what is commonly seen within myeloma. Interestingly, the CD1 and CD2 subgroups appeared vastly different from the remaining subgroups and only exhibited a few changes to their CNV profiles. Although the remaining groups exhibited similar profiles, we identified some interesting profile changes within these subgroups that helped define the structural makeup of each group. One of the more interesting groups observed was the LB subgroup which exhibited gains in chromosomes 2 and 17, which may help account for its standard risk profile. 

Recently, the Mayo Clinic updated the mSMART risk stratification guidelines, in part, by adding an intermediate risk group. Consistent with previous assessments, the high risk group consists of patients whose disease harbors a t(14;16) and t(14;20) translocations (MF subgroup) as well as deletions to 17p and GEP high risk signature. The new intermediate group is defined by hypodiploidy, t(4;14) translocation (MS subgroup) and deletion of chr 13 whereas the standard risk group is defined by the t(11;14) and t(6;14) translocations CD1 and CD2 subgroups and hyperdiploidy (HY and LB subgroups) (Table 1) [26]. To provide a clinical basis to our genetic analysis, we scored our groups based on mSMART guidelines and assigned each subgroup to the appropriate mSMART risk group based on GEP and CytoScan HD data (Table 1). Consistent with previous reports classifying the myeloma subgroups, our analysis identified the MF and PR subgroups as the highest risk followed by the MS and MY subgroups classified in the new intermediate group and finally the CD1, CD2, HY and LB subgroups were determined to be the lowest risk and classified within the standard risk group. 

### 2.4. Chromosomal Positional Enrichment and Pathway Analysis of the Cytogenetically Defined Subgroups Identifies Cytogenetic Driver Lesions and Unique Subgroup Pathway Signatures

The CytoScan HD arrays have recapitulated changes known to occur within myeloma very well as well as identifying genetic profiles unique to individual subgroups. To correlate the changes in gene expression with the CytoScan HD-defined changes we performed chromosomal positional enrichment analysis to identify genes located in regions of chromosome deletions or amplifications where gene expression correlates with CNVs. CNVs determined to have correlative changes with GEPs were further analyzed using Gene Trail and H-Invitational DB Enrichment Analysis Tool (HEAT) to identify regions significantly enriched in MM. The genomic regions identified by Gene Trail and HEAT provide growth advantages to tumor cells when genes within these regions are over-expressed, suggesting that these regions contain “driver” mutations initiating myeloma development and progression and are highlighted in Table 2. 

The regions defined by Gene Trail and HEAT are not surprising, in that they are commonly altered regions in myeloma and include gains to Chr 1q, Chr 3p-3q, Chr 9p-9q, Chr 15q, Chr 19p-19q and deletions to Chr 8p, Chr 13q and Chr 16q. Encoded within these regions are genes involved in chromosomal instability, oncogenesis and drug resistance, which further reinforces the driver characteristic of these genes. 

**Figure 4:**
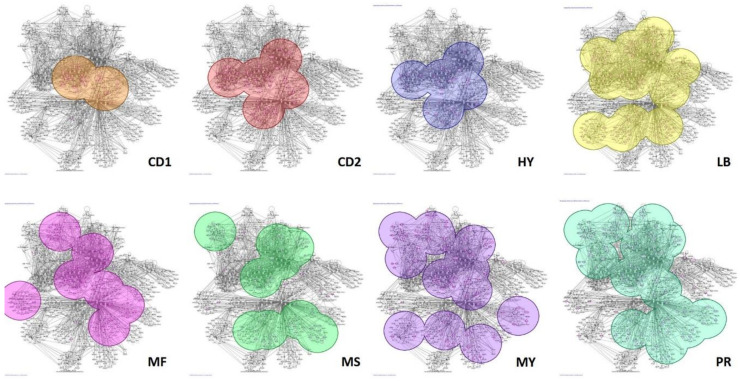
Myeloma Subgroups Exhibit Unique Network Characteristics. Ingenuity pathway analysis was performed on genes identified in GEP and CNV analysis from the 8 myeloma subgroups to identify common and unique protein interaction networks between the subgroups. Results from the analysis identified a common central protein network belonging to all subgroups as well as peripheral networks belonging to only one or a few of the myeloma subgroups. Each network represents the contributions of a single subgroup, highlighted in color, to the overall myeloma network.

The identification of putative driver lesions is an important discovery to the understanding of myeloma oncogenesis and progression, but to fully understand the nuances of myeloma an in-depth analysis of the individual subgroups is warranted. To better understand the differences between the eight myeloma subgroups, Ingenuity pathway analysis was performed on genes identified as significant from the Gene Trail and HEAT analyses. Ingenuity pathway analysis (IPA) allows for the visualization of protein interaction networks that are enriched for within each subgroup. IPA networks also provide a better understanding of the molecular interactions that are occurring within a given tumor. The results from our IPA analysis identified a small network of proteins, highlighted by STAT1 and TCF3, which are enriched for in all eight of the subgroups (Figure 4). Furthermore, FANCL, BAD, RAS and PARP1 are enriched in a large number of the subgroups. Although IPA analysis of shared networks tells a lot about important pathways and molecules in MM in general, one of its more powerful attributes is its ability to differentiate between subgroups and identify protein networks unique to one or a few subgroups. Analysis of individual subgroups identified a few networks unique to a single or two subgroups including PTEN to LB and MY, HNF4A and NF-κB to MF, KRAS and TOR1A to MY and PR and TGFβ to LB and MS subgroups (Figure 4).

### 2.5. Survival and Hazard Ratios from an Independent Cohort Correlate with Results from the TT2 Set

**Figure 5:**
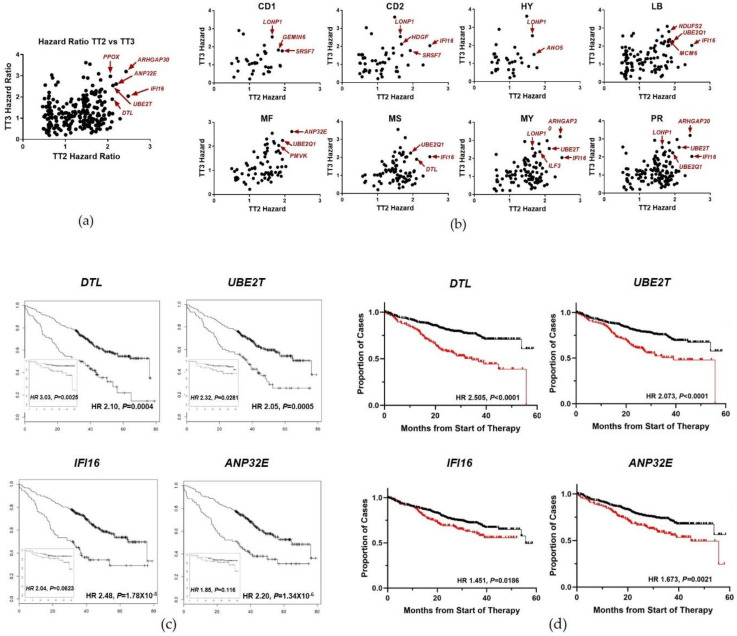
Independent Patient Samples Recapitulate the Results Observed in the Myeloma Expression Model. Hazard and Kaplan-Meier analysis was performed for significantly enriched genes within the TT2 and TT3 cohorts. (**a**) Hazard analysis comparing the complete TT2 and TT3 cohorts. (**b**) Hazard analysis comparing TT2 and TT3 from the individual myeloma subgroups. (**c**) Kaplan-Meier survival analysis for TT2 and TT3 cohorts highlighting select genes identified in the Hazard analysis. Kaplan-Meier and Hazard analysis were performed using the R project 2.14.2. Significance was determined as *p* < 0.05 with an FDR < 0.05 as determined by a student’s *t*-test. (**d**) Kaplan-Meier survival analysis of select genes in CoMMpass cohorts.

To test the reliability of our testing criteria, the TT2 training cohort was compared to an independent myeloma cohort (TT3) to assess the reliability of predicting progression free survival (PFS), overall survival (OS) and hazard ratio (HR), either as a complete cohort or separated into the individual subgroups. To assess the correlation between the two cohorts, HRs were calculated for significantly altered genes for the TT2 and TT3 cohorts. Analysis of the HR for enriched genes demonstrated a strong correlation between the TT2 and TT3 cohorts, with ARHGAP30 (chromosome 1q23), ANP32E (chromosome 1q21), DTL (chromosome 1q32), IFI16 (chromosome 1q21), and UBE2T (chromosome 1q32) exhibiting the strongest correlation of genes analyzed (Figure 5a). Since there was a strong correlation between the two cohorts we wanted to determine if there was a strong correlation between the subgroups as well. HRs for genes enriched in each subgroup were calculated and compared between the two subgroups. Consistent with the results from the TT2 vs TT3 analysis, subgroup comparison exhibited a strong correlation of HRs between the subgroups of TT2 and TT3. Furthermore, ANP32E, ARHGAP30, DTL, UBE2Q1 (chromosome 1q21), UBE2T and IFI16 all exhibit strong correlation between HRs in many of the subgroups (Figure 5b, Table S1). The gene expression of the identified driver genes were analyzed in the subgroups compared to NPC and multiple myeloma cell lines (Figure S1).

The strong HR correlations and the multiple subgroups enriched for DTL, UBE2T, IFI16 and ANP32E prompted us to further analyze these genes. Kaplan-Meier survival analysis was performed for each gene to assess the importance of each gene from the given cohort. The Kaplan-Meier analysis identified the four genes as significant determinants of survival for the TT2 and TT3 cohorts, with the exception of IFI16 for the TT3 cohort (Figure 5c). Interestingly, the survival curves from the TT3 cohort were not as impressive as their counterparts from the TT2 cohort. This observation could be, in part, from the short duration of the TT3 study at the time of analysis. The results from the hazard and Kaplan-Meier analyses suggests that the TT2 and TT3 are well matched and provides further evidence that our new CNV-based model correctly classifies independent myeloma samples. Furthermore, we validated the four genes as significant determinants of MM patient survival in CoMMpass cohort (Figure 5d). It is noteworthy that the genes ANP32E, DTL, IFI16, UBE2Q1, and UBE2T, identified in this unbiased approach, all map to chromosome 1q. These data strongly support previous work from our group and others pointing to a significant role for chromosome 1q gains in conferring an aggressive clinical course in MM [27,28,29,30] and a risk for the conversion of MGUS and smoldering MM to overt MM requiring therapy [31,32,33].

### 2.6. Pathways Enriched within Unique Subgroups Provide a Rationale for Use of Existing Therapies and Development of New Treatments

**Figure 6:**
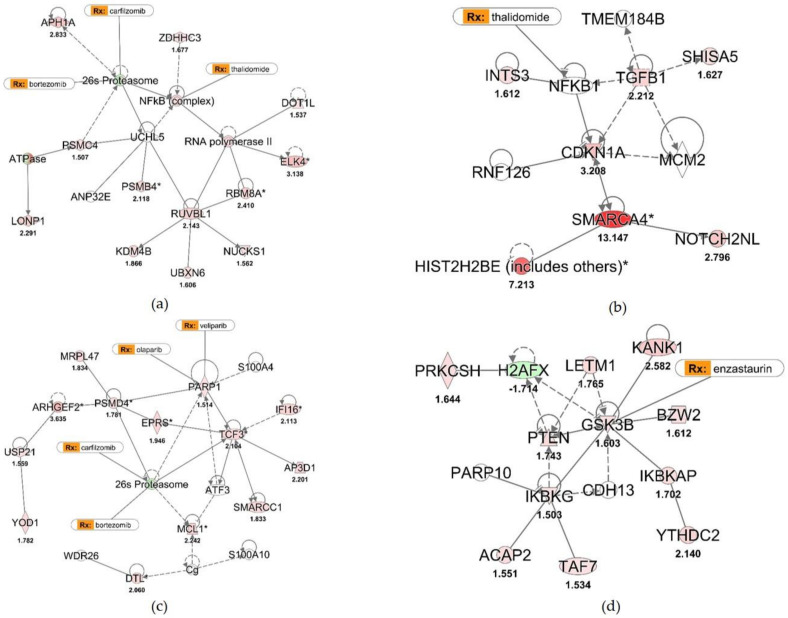
Subgroup Expression Networks are Easily Targeted with Existing Therapeutics. In-depth analysis of protein networks, unique to individual or a small number of myeloma subgroups, to identify novel therapeutic regiments for myeloma. (**a**) NF-κB, ANP32E and proteasome protein network unique to the MF subgroup. (**b**) NF-κB and TGFβ protein network unique to the MF subgroup. (**c**) PARP1 and 26S proteasome network unique to the MS, MY and PR subgroups. (**d**) GSK3B, PARP10 and PTEN protein network unique to the MY and LB subgroups.

We have shown that we can differentiate subgroups based on their protein network signature and each subgroup displays a pattern unique to that subgroup making it possible to identify each subgroup based on this unique signature. A novel and useful approach to this type of analysis is the ability to predict therapeutic efficacy and identify novel therapeutics based on an individual’s subgroup classification. For example, the MF subgroup is the only subgroup enriched for the NF-κB, RNA polymerase II, 26s proteasome network (Figure 4 MF network and Figure 6a) and the NF-κB ANP32E network (Figure 4 MF network and Figure 6b). NF-κB is targeted by thalidomide suggesting that patients within this group may respond better to treatment regimens including thalidomide. Furthermore, the network in Figure 6b includes the protein phosphatase 2A inhibitor ANP32E. Hazard analysis identified ANP32E as an important risk predictor both within the total cohort and the MF subgroup analysis, suggesting that the addition of an ANP32E inhibitor to a thalidomide-based regiment may 62improve drug efficacy in patients whose tumors have 1q gains and elevated ANP32E. 

Additional analysis identified the PARP1 network as important for the pathogenesis within the MS and potentially the MY and PR subgroups. An important and tangible finding within this network was the identification of two known PARP inhibitors: olaparib and veliparib (Figure 4 MS network and Figure 6c). This discovery has important therapeutic implications. The MS subgroup often harbors amplification of chr 1q and deletion of chr 13q, suggesting that it would be classified as an intermediate risk myeloma and mSMART guidelines would suggest a treatment regimen containing bortezomib. Furthermore, the proteasome network has connections to the PARP1 network, suggesting a treatment regimen containing bortezomib and olaparib or veliparib would potentially provide additional benefits to patients within the MS subgroup. The addition of a PARP inhibitor to treatment regimens may also benefit patients within the LB subgroup as well. The MY and LB subgroups have enrichment of the GSK3B, PTEN and PARP10 network (Figure 4 MY and LB networks and Figure 6d). One of the PARP inhibitors along with the PTEN/AKT/mTOR inhibitor enzastaurin may provide added benefit to patients within the MY and LB subgroups. Although the treatment benefits highlighted above are still theoretical, the accuracy of our prediction model suggests that treatment with select inhibitors along with current therapeutics is warranted, especially in high risk MM experiencing little benefit from current regimens.

## 3. Discussion

In this report we demonstrate the heterogeneity within myeloma through gene expression and copy number analysis. Consistent with other reports, we show that the complexity of the disease increases as the disease progresses, with MGUS exhibiting the least amount of abnormalities and MM the most. Furthermore, we generated eight unique subgroups based on GEP and CNV data and show that molecular and CNV subgroups exhibit dramatically different expression and chromosome CNV profiles. Interestingly, Ingenuity pathway analysis of the subgroups revealed significant overlap of pathways; however, each subgroup was enriched for a number of unique pathways. Further understanding of the pathway signature may inform implementation of novel treatment regimens using currently available treatments and development of therapeutics designed specifically for the individual subgroups.

Recent work by Vogelstein et al. estimates the number of somatic mutations in cancer to vary greatly depending on the tumor type [34]. They identify blood cancers, such as acute myeloid leukemia, as some of the least complex with an average of 10–15 somatic mutations per sample, whereas colorectal and lung carcinomas and melanomas are considered to be some of the most complex with hundreds of mutations per sample [34]. Although the number of mutations per cancer varies greatly, a majority of the mutations are considered to be “passenger mutations”, or mutations that are not thought to confer a growth advantage to the tumor. Whereas a small subset of the mutations, “driver mutations”, do confer a growth advantage, it is the accumulation of driver mutations that result in the onset of clinical cancer. Interestingly, 125 driver mutations have been identified in cancer, with a majority of the mutations occurring in tumor suppressor genes. Of the driver mutations identified, protein kinases make up an appreciable number and have been validated as novel therapeutic targets [35]. Furthermore, additional driver mutations such as in MYC, BCL2, MMSET and FGFR3 are considered as important driver mutations in the development of cancer [36,37,38,39].

In the current study we set out to identify CNV changes representing driver mutations in myeloma. Interestingly, many changes identified as cytogenetic drivers are commonly observed abnormalities, such as gains to chromosomal regions 1q, 3p26-3q39, 9p24–9q34, 15q11–q26 and 19p13–19q13 and losses to chromosomal regions 8p, 13q and 16q (Table 2). Indeed, ANP32E, DTL, IFI16, UBE2Q1, and UBE2T genes linked to a high hazard of death, identified in this study, all map to chromosome 1q. Strongly supporting previous work from our group and others pointing to a significant role for chromosome 1q gains in conferring an aggressive clinical course in MM [27,28,29,30] and a risk for the conversion of MGUS and smoldering MM to overt MM requiring therapy [31,32,33].

Interestingly, results from our analysis are consistent with the findings from Greenman et al., suggesting protein kinases represent an important fraction of driver mutations [35]. We identified numerous protein kinases altered within our diver CNV regions, including known kinase regulators like CKS1B and actual kinases like NEK2, which have been linked to poor clinical outcomes within MM [40,41]. Furthermore, numerous other protein kinases implicated as driver genes in various other cancers were identified here, including NTRK3 and MAP2K7 [35,42,43].

In the current study we stove to better understand the molecular heterogeneity that exists within myeloma patients. Interestingly, we only discovered a small network of proteins consistent across all eight myeloma subgroups, highlighted by the over-expression of STAT1, TCF3 and interferon alpha. Furthermore, there were fewer subgroup unique networks than anticipated. One subgroup that exhibited a number of unique subgroups was the MF subgroup. The MF subgroup exhibited two unique protein networks that centered on the increased expression of NF-κB. The more interesting of the two networks is enriched with RNA polymerase II, the 26S proteasome and ANP32E. As demonstrated above, ANP32E has a strong correlation with survival and its overexpression results in a decreased PFS and OS (Figure 5a and data not shown). ANP32E has been shown to play a role in chromatin remodeling and regulate transcription and may contribute to the increased cellular transcriptional dysregulation in myeloma [44]. Although the number of subgroup specific protein networks was small, the analysis provided some interesting discoveries in regard to the similarities and differences between the subgroups. Many of the subgroups are associated with increased in NF-κB, IFNβ, P38/MAPK and the proteasome network. Furthermore, JUNB and ATF3 are enriched in a few of the subgroups.

The model highlighted here within demonstrates the ability to accurately classify myeloma and identify important pathways involved in transcription regulation, apoptosis and oncogenesis however, an important characteristic of this model is its ability to identify novel drug targets unique to a specific subgroup or common among all of the subgroups. We have highlighted a few known inhibitors that may improve outcomes of patients within subgroups. Future care for myeloma patients will involve the classification of the type of myeloma followed by use of a classification-informed personalized treatment regimen.

## 4. Materials and Methods 

### 4.1. Patient Samples

Clinical bone marrow samples were obtained from MM patients in Huntsman Cancer Institute, University of Utah. CD138+ cells were isolated from MM patients’ bone marrow using autoMACS and CD138 microbeads (Miltenyl Biotec, Bergisch Gladbach, Germany).

### 4.2. Chromosomal Microarray Analysis

Hybridization-based genomic profiling arrays were performed in a blinded fashion using the CytoSan HD array platform having 1.8 million and 2.6 million combined SNP and CNV markers with the median inter- marker distance of 500–600 bases. (Affymetrix, Inc., Santa Clara, CA, USA). Hybridizations were performed according to the manufacturer’s protocols. CEL files obtained by the CytoScan HD array platform were analyzed using the Chromosome Analysis Suite software package (Affymetrix), and the Nexus copy number software (Biodiscovery Inc., Hawthorne, CA, USA) using annotations of genome version GRCh37 (hg19). Only those achieving the manufacturer’s quality cut-off measures were included in the analysis.

### 4.3. Gene-Expression Profiling Analysis

Gene expression profiles for 351 patients with MM, 22 patients with NPC, 44 with MGUS, and 12 with SMM were performed using the Affymetrix U133Plus2.0 microarray as previously described [17]. Microarray data used in this study have been deposited in the NIH Gene Expression Omnibus under accession numbers GSE2658 and GSE5900. Gene expression analysis was performed using Partek Genomic Suite 6.6 with fold changes calculated as relative changes compared to normal plasma cells, with fold-changes of at least 1.5 fold with a *p* < 0.05 and a FDR < 0.05 were considered for further analysis. 

### 4.4. Patient Survival and Hazard Analysis

Kaplan-Meier survival analysis was performed using the R project version 2.14 (http://cran.r-project.org/). Patient samples were divided into quartiles with the highest quartile plotted against the lower three quartiles. Hazard analysis was performed in the R projects survcomp 1.1.6 survival package using the Cox proportional-hazards regression model. Significance was determined as *p* < 0.05.

## 5. Conclusions

Myeloma is a genetically heterogeneous disease that can be classified to one of a small number of molecularly defined subgroups. This study was designed to answer a few questions geared at improving our understanding of cytogenetic changes resulting in the development and progression of myeloma. We identified eight cytogenetic driver lesions essential to development and progression of myeloma highlighted by the amplification of chromosome 1q. Furthermore, our cytogenetic analysis along with gene expression arrays advanced our understanding of the subgroups and show that there are a small number of protein networks common to all subgroups. Moreover, we identified a well-defined protein network signature for each subgroup that can be predicted accurately through the use of CNV arrays, potentially eliminating the need for GEP arrays and large FISH panels clinically. Finally, we identified novel therapeutic targets unique to specific subgroups that can work in concert with existing therapies to lessen the severity of myeloma and extend overall survival. Through additional understanding of the modelling system presented here additional drug targets will be identified for each subgroup and help lead to personalized treatment regimens for patients, irrespective of the myeloma classification.

## Figures and Tables

**Table 1:** MM mSMART Risk Stratification.

High Risk	Intermediate Risk	Standard Risk
FISH	FISH	All others including
t(14;16)	t(4;14)	FISH
t(14;20)	Cytogenetic del 13	t(11;14)
Cytogenetic Del 17p	Hypodiploidy	t(6;14)
GEP	PCLI ≥ 3%	
High risk signature		
**GEP Defined Molecular Subgroup Classification**		
MF	MS	CD1
PR	MY	CD2
		HY
		LB

Risk stratification for active myeloma as determined by the Mayo Clinic mSMART consensus guidelines for 2013 [14]. PCLI= Plasma cell labeling index. Subgroups are assigned to risk groups based on cytogenetic markers and classification systems previously developed [13,23]. Table adapted from Mikhael et al. [14].

**Table 2:** Cytogenetic Drivers in MM.

Gains	Deletions
1q21.1-1q44	8p23.3-8p11.1
3p26.3-3q29	13q11-13q34
9p24.3-9q34.3	16q11.1-16q24.3
15q11.2-15q26,3	
19p13.1-19q13.43	

CytoScan HD and gene expression profile analysis identified cytogenetic gains and deletions as drivers of oncogenesis and disease progression in myeloma. Consistent with previous findings, the cytogenetic regions contain known neoplastic drivers common to many known cancer types, including CKS1B, NEK2, NTRK3 and MAP2K7.

## Data Availability

The data presented in this study are openly available in the NIH Gene Expression Omnibus under accession number GSE164554.

## Supplementary Materials

The following are available online at https://www.mdpi.com/2072-6694/13/3/517/s1, Table S1: Correlation between the TT2 and TT3 cohorts. Figure S1: Gene expression of the four identified driver genes in molecular subgroups.
