# Supplementary material for: Genetic Analysis of Multiple Myeloma Identifies Cytogenetic Alterations Implicated in Disease Complexity and Progression
Source: Cancers (Basel). 2021 Jan 29;13(3):517. doi: 10.3390/cancers13030517 (PMC7866300; doi:10.3390/cancers13030517)
Supplement: Supplementary file 1 [file cancers-13-00517-s001.pdf]

cancers

Supplementary materials

Genetic Analysis of Multiple Myeloma Identifies Cytogenetic Alterations Implicated in Disease Complexity and Progression

Can Li [1,2,†], Erik B. Wendlandt [3,†], Benjamin Darbro [4], Hongwei Xu [1], Gregory S. Thomas [3], Guido Tricot [1], Fangping Chen [2], John D. Shaughnessy, Jr. [1] and Fenghuang Zhan [1,*]

1 Myeloma Center, Department of Internal Medicine, Winthrop P. Rockefeller Cancer Institute, University of Arkansas for Medical Sciences, Little Rock, AR 72205, USA; HXu@uams.edu (H.X.); GJTricot@uams.edu (G.T.); JDShaughnessy@uams.edu (J.D.S.J.)
2 Department of Hematology, Xiangya Hospital, Central South University, Changsha 410008 China; cli2@uams.edu (C.L.); xychenfp@csu.edu.cn (F.C.)
3 Department of Internal Medicine, University of Iowa, Iowa City, IA 52242, USA; ewendlandt@idtdna.com (E.B.W.); gregory-thomas@uiowa.edu (G.S.T.)
4 Cytogenetics and Molecular Laboratory, Carver College of Medicine, University of Iowa, Iowa City, IA 52242, USA; benjamin-darbro@uiowa.edu
* Correspondence: FZhan@uams.edu
† These authors contributed equally to this work.

Citation: Li, C.; Wendlandt, E.B.; Darbro, B.; Xu, H.; Thomas, G.S.; Tricot, G.; Chen, F.; Shaughnessy, J.D., Jr.; Zhan, F. Genetic Analysis of Multiple Myeloma Identifies Cytogenetic Alterations Implicated in Disease Complexity and Progression. *Cancers* **2021**, *13*, 517. https://doi.org/10.3390/cancers13030517

Received: date
Accepted: date
Published: 29 January 2021

Publisher's Note: MDPI stays neutral with regard to jurisdictional claims in published maps and institutional affiliations.

Copyright: © 2021 by the authors. Licensee MDPI, Basel, Switzerland. This article is an open access article distributed under the terms and conditions of the Creative Commons Attribution (CC BY) license (http://creativecommons.org/licenses/by/4.0/).

Supplementary materials

Table S1. Correlation between the TT2 and TT3 cohorts.

Subgroup	r^2	Slope
CD1	0.0035	y = 0.0956x + 1.1095
CD2	0.0394	y = 0.2896x + 0.9152
HY	0.0296	y = 0.3348x + 0.9085
LB	0.0713	y = 0.3553x + 0.915
MF	0.2041	y = 0.6034x + 0.354
MS	0.0373	y = 0.3265x + 0.7612
MY	0.1232	y = 0.5178x + 0.4635
PR	0.0819	y = 0.4078x + 0.6326

Correlation of gene expression intensity between the TT2 and TT3 cohorts in 8 subgroups from Figure 5b.

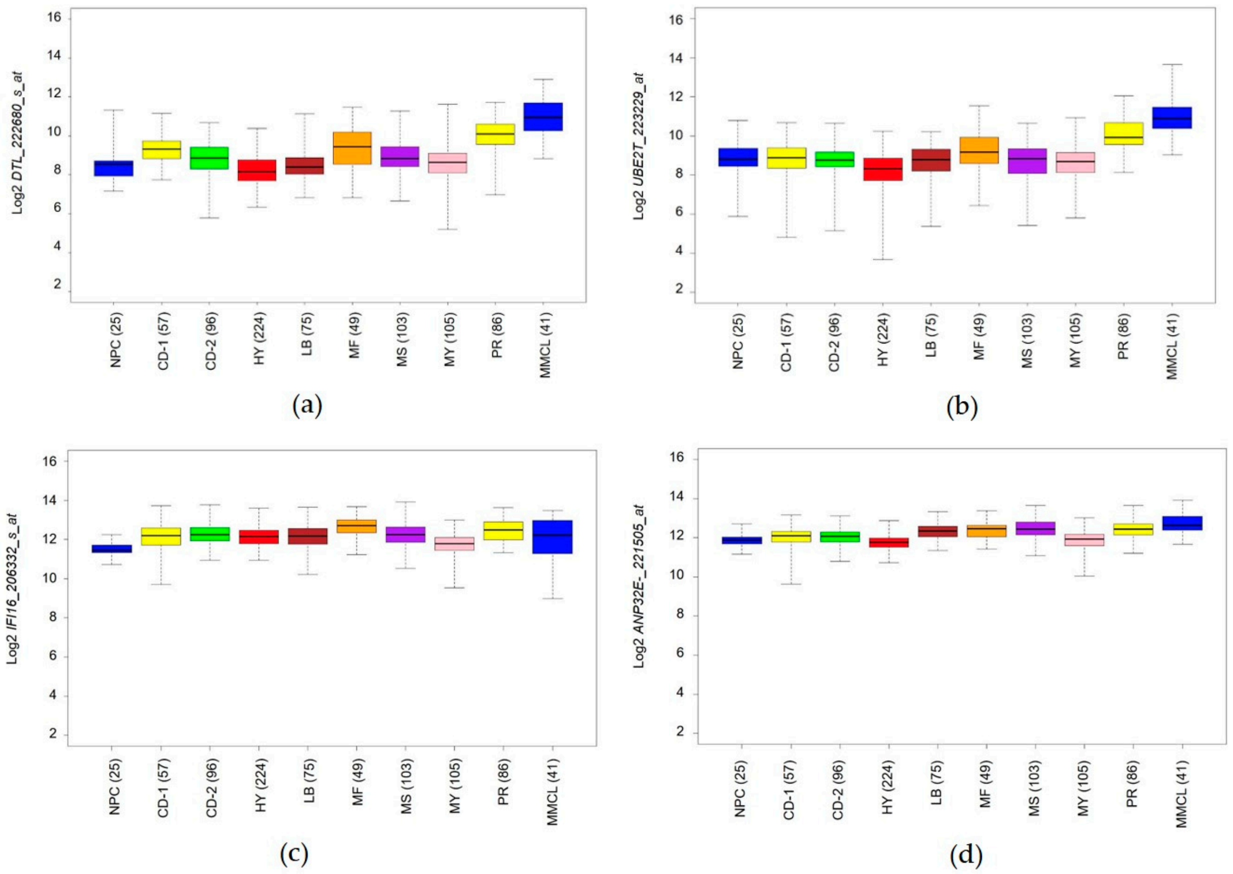

Figure S1. Gene expression of the four identified driver genes in molecular subgroups.Boxplots of the MAS5.0 transformed Affymetrix Signal for the four identified driver genes (a) DTL (b) UBE2T (c) IFI16 and (d) ANP32E in CD138-selected plasma cells isolated from bone marrow aspirates from healthy donors (NPC) and newly diagnosed MM (CD-1, CD-2, HY, LB, MF, MS, MY and PR). Also included are expression values from multiple myeloma cell lines (MMCL). The number of cases represented in each box plot is indicated by the number in parentheses above the sample identifier.
